# Evaluation of cognitive functions of children aged 8-18 years with acute leukemia 2 years after the end of treatment

**DOI:** 10.1192/j.eurpsy.2025.1114

**Published:** 2025-08-26

**Authors:** S. Çetin, A. Akay, H. Ören

**Affiliations:** 1 Child and Adolescent Psychiatry Department; 2 Pediatric Hematology Department, Dokuz Eylül University Faculty of Medicine, İZMİR, Türkiye

## Abstract

**Introduction:**

Children with a history of acute leukemia may experience some deficits in their cognitive functions in the late period (Hewitt *et al.* Childhood Cancer Survivorship 2003).These deficits negatively affect their quality of life by impairing their academic, social and psychological functioning (Jacola *et al.* Lancet Psychiatry 2016; 3-10-965-972).

**Objectives:**

The aim of this study was to compare the cognitive functions of children with a history of leukemia compared to their healthy peers.

**Methods:**

Our study was conducted with 42 cases with a history of leukemia who were followed up in the Pediatric Hematology outpatient clinic of Dokuz Eylül University Faculty of Medicine and 41 healthy controls who were admitted to the Child and Adolescent Psychiatry outpatient clinic of Dokuz Eylül University Faculty of Medicine.After obtaining informed consent from both parents and participants, the Wisconsin Card Sorting Test (WCST), Stroop Test-TBAG form, Trail Making Test (TMT) A and B forms were administered to all participants.The parents of all participants were given a sociodemographic data form and the Childhood Behavior Checklist (CBCL) and asked to complete it.The teachers of all participants were given the Conner’s Teacher’s Rating Scale-Revised Long form (CTRS-RL) and asked to complete it.

**Results:**

All sub-dimension CBCL scores of the case group were significantly higher than the control group (p<0.05).All sub-dimension CTRS scores of the case group were found to be significantly higher than the control group.The case group performed worse than healthy controls in WCST, Stroop-TBAG and Trail-Making Test. The mean Stroop2-time score was higher in the group that received cranial radiotherapy, whereas the Trail-Making-B-error score was higher in the group that did not.The group receiving the medium-high risk chemotherapy protocol performed worse overall on the Stroop test.Women in the case group performed worse on the WCST than men.Stroop5-error scores were higher in the group with a history of sequelae.It can be said that there is a negative correlation between age at diagnosis, age at the end of treatment and time elapsed after treatment and cognitive test performances.There was a moderate positive correlation between the duration of the treatment and the CBCL-attention problems score (r=0.348, p=0.024) and the CTRS-attention problems score (r=0.432, p=0.04).

**Conclusions:**

In conclusion, it can be said that leukemia treatment may cause deficits in areas such as attention, memory, learning, processing speed, executive function and problem solving in the late period (Montour-Proulx *et al.* J Child Neurol 2005; 20 2 129-133).The difference in the severity of deficits in these areas between individuals can be explained by many biological, social and psychological factors (Duffner *et al.* J Pediatr Hematol Oncol 2014; 36 1 8-15).

**Disclosure of Interest:**

None Declared

